# Editorial

**Published:** 1844-11-16

**Authors:** 


### THE MEDICAL EXAMINER.

PHILADELPHIA, NOV. 16, 1844.

### MEDICAL CLASSES.

Of the number of students in attendance on the lectures in the various Medical Colleges throughout the country, we are not yet informed. In Philadelphia, the size of the class at the University of Pennsylvania is above the average of that ancient school, while that of Jefferson Medical College is greater than at any former period. We learn also, that the Pennsylvania College is doing better than last year. Of those in neighbouring cities, reports are contradictory, but it is probable they are not materially below their general average.

---

### INSANE IN CANADA.

It is gratifying to perceive the growing interest felt in the welfare of those afflicted with the greatest of all deprivations—the loss of reason. We have had repeated occasion to notice the progress of various institutions for the relief of such in different parts of the United States, and it affords us pleasure to learn that the government of Canada has taken effective measures to secure like advantages to the objects of such establishments in Canada. A law has been enacted for the erection of an extensive building at Toronto; seventy-five acres of ground have been purchased for its site, within the bounds of the City, and a Board of Commissioners appointed to carry the plan into effect. Two members of that body, viz. Professor King and Mr. Cheward, are now making a visit to the principal institutions in this country, with the view of obtaining information on the subject committed to their care. This is beginning right; instead of acting first and looking afterward, as some do. From our knowledge of the gentlemen deputed to make this survey, we anticipate a thorough investigation, and an able report.

---

### CLINICAL REPORTS.

In our present number, we commence our reports of clinical lectures to be delivered during the winter at the Philadelphia Institutions. We have taken measures to be regularly furnished with reports of some of the courses, and shall be glad to be supplied with *all.* It is our wish to make the clinical opportunities afforded by Philadelphia more extensively known, that our brethren at a distance, who are unable to visit the place, may know something of what is doing among us.

---

### DEATH OF DR. FORRY.

From the New York Herald we learn that Dr. Samuel Forry, editor of the New York Journal of Medicine, died in that city on Saturday last.

Dr. Forry, we believe, was a native of Lancaster county, Pennsylvania. He graduated in the Jefferson Medical College in 1832, after which he was attached to the U. S. army, and served some time in Florida as assistant surgeon. Although he died at the early age of thirty-three, he had already distinguished himself by his zealous and successful labors in the promotion of science.

